# Some Account of the Good Effects of the Gastric Juice in the Cure of Ulcers; and of Magistery of Bismuth in Painful Affections of the Stomach
*M. Senebier, of Geneva, seems to have been the first who suggested the use of the gastric juice as a topical application. In a paper on this subject, entitled "Observations "importantes sur l'usage du suc gastrique dans la Chirurgie," inserted in the Journal de Physique for March, 1785, he relates several cases in which it had been tried, at his request, by M. Jurine, Surgeon at Geneva; and, from its effects in those cases, supposes it to possess properties similar to those ascribed to it by M. Butini.—Numerous experiments of the same kind, and with similar success, it is said, have likewise been made by Sig. Carminati, Professor of Surgery at Pavia; but at the Westminster Hospital, where this new remedy has been tried in several cases of ill-conditioned ulcers by Mr. Watson, its good effects, as he has been pleased to inform us, have not been sufficiently obvious to induce him to persevere in its use.—Editor.


**Published:** 1785

**Authors:** 


IX. *Some Account of the good Effects of the Gaftric Juice* in the Cure of Ulcers; and of Magiftery of Bifmuth in painful Affections of the Stomach. Communicated in a Letter to Dr.* Simmons *by M.* Butini, *M. D. Phyfician at Geneva.*

TWO remedies have of late been much employed here, of which you will, perhaps, not be forry to have fome account.

1. *The*

* M. Senebier, of Geneva, feems to have been the firft who fuggefted the ufe of the gaftric juice as a topical application

1. *The Gastric Juice.* — The use of this has been adopted in the cure of obstinate ulcers with the greatest success. No external application has a better effect than this in cleansing the surface of ulcers, correcting their fœtor, or disposing them to cicatrise. I am now trying it in the case of an elderly man, who has a phagedænic ulcer on his left cheek, which began about fifteen years ago, and for several years past has been continually spreading, in spite of a variety of remedies, and the endeavours of our most experienced surgeons to heal it. When I first saw him the ulcer was several inches in diameter, with hard, prominent tion. In a paper on this subject, entitled "*Observations* "*importantes sur l'usage du suc gastrique dans la Chirurgie*," inserted in the *Journal de Physique* for March, 1785, he relates several cases in which it had been tried, at his request, by M. Jurine, Surgeon at Geneva; and, from its effects in those cases, supposes it to possess properties similar to those ascribed to it by M. Butini. — Numerous experiments of the same kind, and with similar success, it is said, have likewise been made by Sig. Carminati, Professor of Surgery at Pavia; but at the Westminster Hospital, where this new remedy has been tried in several cases of ill-conditioned ulcers by Mr. Watson, its good effects, as he has been pleased to inform us, have not been sufficiently obvious to induce him to persevere in its use. — EDITOR.

edges, edges, and had ſpread almoſt to the mouth. In the ſpace of four weeks the application of gaſtric juice, aſſiſted by the internal uſe of bark and corroſive ſublimate, has produced the moſt favourable alteration imaginable in the edges and complexion of the ulcer, and has reduced it to a third of its former extent.—The gaſtric juice is taken from the ſtomach of a ſheep that has been kept a day without food. The butcher who ſupplies it furniſhes us with freſh juice every day. A doſſil of lint moiſtened with it is applied to the ulcer, and renewed eight times in the day.

2. *Magiſtery of Biſmuth.* — This remedy may be conſidered almoſt as a ſpecific in chronic and nervous pain of the ſtomach, a complaint very frequent here, and hitherto very difficult to cure. Numerous trials have been made of its effects in ſuch caſes, and, in general, with the beſt ſucceſs. The doſe at firſt is a grain four times a day, which is gradually increaſed, and, in ſome caſes, to a drachm or more. It is ſo far from exciting nauſea, like the flowers of zinc, that, in the greater number of patients, it ſeems to prevent ſickneſs.

*Geneva,*
October 30, 1785.

SECTION

